# A Biopsychosocial Overview of Speech Disorders: Neuroanatomical, Genetic, and Environmental Insights

**DOI:** 10.3390/biomedicines13010239

**Published:** 2025-01-20

**Authors:** Diya Jaishankar, Tanvi Raghuram, Bhuvanesh Kumar Raju, Divyanka Swarna, Shriya Parekh, Narendra Chirmule, Vikramsingh Gujar

**Affiliations:** 1 Department of Microbiology, University of California, San Diego, CA 92093, USA; dijaishankar@ucsd.edu; 2 Advancement and Research in the Sciences and Arts (ARISA) Foundation, Pune 411045, India; tanvi.raghuram@arisafoundation.org (T.R.); divyankaswarna@gmail.com (D.S.); shriya.parekh@arisafoundation.org (S.P.); 3 Department of Anatomy & Cell Biology, Oklahoma State University Center for Health Sciences, Tulsa, OK 74107, USA; braju@okstate.edu; 4 SymphonyTech Biologics, Philadelphia, PA 19143, USA

**Keywords:** speech disorders, neuroanatomy, genetics, environmental factors, language pathophysiology, comorbidities, stuttering

## Abstract

Speech disorders encompass a complex interplay of neuroanatomical, genetic, and environmental factors affecting individuals’ communication ability. This review synthesizes current insights into the neuroanatomy, genetic underpinnings, and environmental influences contributing to speech disorders. Neuroanatomical structures, such as Broca’s area, Wernicke’s area, the arcuate fasciculus, and basal ganglia, along with their connectivity, play critical roles in speech production, comprehension, and motor coordination. Advances in the understanding of intricate brain networks involved in language offer insights into typical speech development and the pathophysiology of speech disorders. Genetic studies have identified key genes involved in neural migration and synaptic connectivity, further elucidating the role of genetic mutations in speech disorders, such as stuttering and speech sound disorders. Beyond the biological mechanisms, this review explores the profound impact of psychological factors, including anxiety, depression, and neurodevelopmental conditions, on individuals with speech disorders. Psychosocial comorbidities often exacerbate speech disorders, complicating diagnosis and treatment and underscoring the need for a holistic approach to managing these conditions. Future directions point toward leveraging genetic testing, digital technologies, and personalized therapies, alongside addressing the psychosocial dimensions, to improve outcomes for individuals with speech disorders. This comprehensive overview aims to inform future research and therapeutic advancements, particularly in treating fluency disorders like stuttering.

## 1. Introduction

Speech disorders are manifested due to dysfunctions in neuroanatomy, genetics, and the environment [1,2]. Neuroanatomical aspects of speech are orchestrated by a dynamic interplay between various brain regions, including Broca’s area and Wernicke’s area, which are essential for language production and comprehension, respectively [3]. Functional imaging techniques, such as fMRI, have unveiled the neural circuits that activate during speech tasks, revealing the intricate choreography of motor planning, auditory feedback, and syntactic processing [4]. Genetic studies have identified key genes involved with neural migration and synaptic connectivity that are associated with language development [5]. The interaction between genetics and neuroscience of normal speech could lead to a deeper comprehension of language disorders and potential interventions. We have reviewed advances in speech disorders, the impact of comorbidities of neurogenic disorders, and psychological influences on this disorder. This review aims to understand the advances in the field, inform the next phase of a study, and evaluate the progress in therapies for stuttering. We plan to conduct surveys and interviews of speech therapists who treat patients with speech disorders.

## 2. Neuroanatomy of Speech

**Figure 1:**
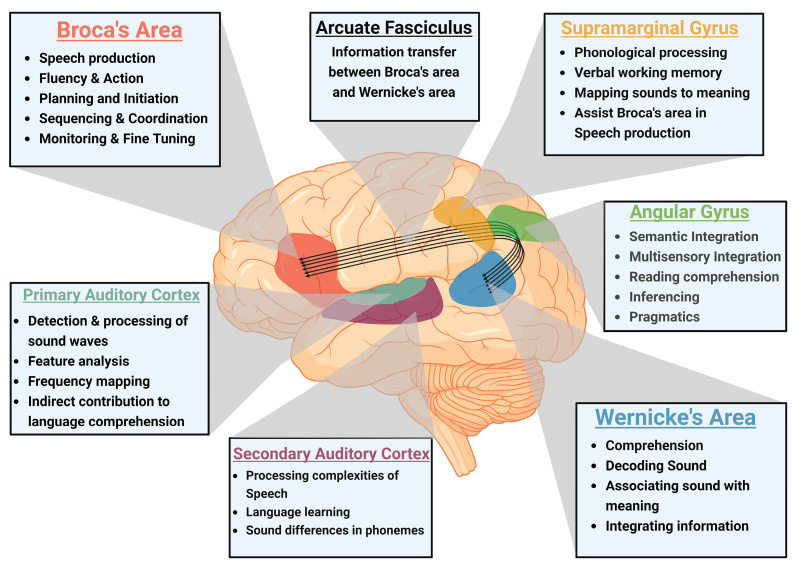
Neuroanatomy of language processing and speech production: The figure emphasizes the interconnected nature of Broca’s area, Wernicke’s area, and Arcuate Fasciculus in speech production and comprehension. It also illustrates supramarginal gyrus and angular gyrus that are involved in phonological processing and semantic integration, along with the primary and secondary auditory cortex responsible for auditory processing (figure created using BioRender).

Speech development is a multifaceted process that begins in infancy and continues through early childhood, with the brain undergoing significant structural and functional changes to support this process. The neuroanatomy of speech development is deeply embedded in the dynamic interactions between various cortical and subcortical regions and the integration of sensory feedback with motor control (Figure 1) [6]. This extended overview explores these connections in greater detail.

### 2.1. Neuroanatomical Structures Involved in Speech Development

#### 2.1.1. Broca’s Area: Motor Aspects of Speech Production

Broca’s area (Brodmann areas 44 and 45), located in most individuals’ left inferior frontal gyrus, is critical for the motor aspects of speech production. This region is responsible for generating the motor plans required for fluent speech and the complex syntactic structuring of sentences [7]. The role of Broca’s area extends beyond mere speech production—it is also involved in aspects of language comprehension that require processing of complex syntax, such as understanding sentences with non-canonical word orders [8]. Broca’s area undergoes gradual maturation during speech development, closely tied to the child’s increasing ability to produce coherent speech. Functional MRI studies have shown that activation in this region becomes more specialized for language production as children transition from babbling to producing meaningful words and sentences [9].

#### 2.1.2. Wernicke’s Area: Language Comprehension

Wernicke’s area, located in the posterior part of the superior temporal gyrus, is primarily responsible for language comprehension. This region is heavily involved in processing auditory information, particularly the recognition of phonemes and words. The development of Wernicke’s area parallels a child’s growing ability to understand spoken language, evident in the early stages of speech development when infants begin to recognize familiar words and respond to spoken commands [10]. Neuroimaging studies have demonstrated that Wernicke’s area and adjacent auditory cortex regions are highly active when children listen to spoken language. As language comprehension skills develop, the connectivity between Wernicke’s area and other language-related regions, such as Broca’s area, strengthens, facilitating more efficient communication between language production and comprehension networks [11].

#### 2.1.3. Arcuate Fasciculus: Connectivity and Coordination

The arcuate fasciculus, a white matter tract connecting Broca’s and Wernicke’s areas, is crucial in integrating speech production and comprehension. It is essential for repeating words and sentences and tasks that require auditory feedback, such as mimicking sounds or learning new words. Damage to the arcuate fasciculus can result in conduction aphasia, where individuals struggle to repeat spoken words despite having intact comprehension and speech production abilities [12]. During early childhood, the arcuate fasciculus undergoes significant myelination, enhancing communication speed and efficiency between the frontal and temporal language regions. This process is vital for developing phonological working memory, a key component of language acquisition that allows children to hold and manipulate sound patterns in their minds while learning to speak [13].

#### 2.1.4. Basal Ganglia: Modulation of Motor Speech

The basal ganglia, a group of subcortical nuclei, are involved in the modulation of motor activities, including speech. These structures regulate speech timing, rhythm, and the smooth execution of articulatory movements. Dysfunction in the basal ganglia is associated with motor speech disorders, such as dysarthria, where speech becomes slurred or slow [14]. In speech development, the basal ganglia work with cortical motor areas to refine the motor commands necessary for clear and fluent speech. Research suggests that these structures also play a role in acquiring language skills, particularly in procedural memory, which supports learning speech patterns and grammar rules through repeated practice [15].

#### 2.1.5. Cerebellum: Coordination and Timing

The cerebellum, traditionally associated with motor coordination and balance, also contributes to speech development by ensuring the precise timing and smooth execution of speech movements. The cerebellum interacts with cortical speech regions to fine-tune the motor commands for articulation, helping to coordinate the rapid and intricate movements of the mouth, tongue, and vocal cords required for fluent speech [14]. Functional neuroimaging studies have shown that the cerebellum is activated during speech production tasks that require precise motor control, such as speaking rapidly or articulating complex sentences. The cerebellum’s role in speech is also evident in developmental disorders like ataxic dysarthria, where cerebellar dysfunction leads to difficulty coordinating speech movements [16].

#### 2.1.6. Primary Motor Cortex: Execution of Speech Movements

The primary motor cortex, particularly the area responsible for controlling the mouth, face, and tongue muscles, is crucial for the physical execution of speech. This region receives input from the premotor cortex and sensory cortices to generate the motor commands for articulate speech [17]. In speech development, the primary motor cortex works closely with subcortical regions like the basal ganglia and cerebellum to refine speech motor control. As children practice speaking, the neural circuits in the motor cortex are fine-tuned to produce smooth and coordinated speech. This is supported by feedback from sensory systems, which helps adjust motor outputs based on auditory and tactile sensations [18].

### 2.2. Neurodevelopmental Changes in Speech and Language Acquisition

During infancy and early childhood, the brain undergoes critical periods of plasticity, where it is particularly receptive to language input. During these periods, the language networks in the brain are shaped by exposure to spoken language, leading to the development of neural circuits that support speech production and comprehension [19]. For instance, infants are born able to distinguish between the phonemes of all human languages. Still, by the end of the first year, their brains become specialized for the phonemes of their native language, primarily influenced by the environment in which they are raised [20]. This specialization reflects changes in the connectivity and functionality of brain regions involved in speech and language processing. The maturation of white matter tracts, such as the arcuate fasciculus, is essential during this period, as it supports the integration of auditory and motor systems necessary for speech development [13]. Similarly, the myelination of other speech-related pathways continues throughout childhood, contributing to refining language skills, including vocabulary expansion and the ability to construct complex sentences.

The neuroanatomy of speech development is a dynamic and interconnected system that involves several key brain regions, including Broca’s and Wernicke’s areas, the arcuate fasciculus, the basal ganglia, the cerebellum, and the primary motor cortex. These structures work together to support the intricate processes of speech production, language comprehension, and motor coordination, which are crucial for successful speech development. The interplay between these regions and the continuous refinement of neural circuits highlights the brain’s remarkable plasticity during early language acquisition, shaping the foundations for lifelong communication skills.

## 3. Genetics of Speech

**Figure 2:**
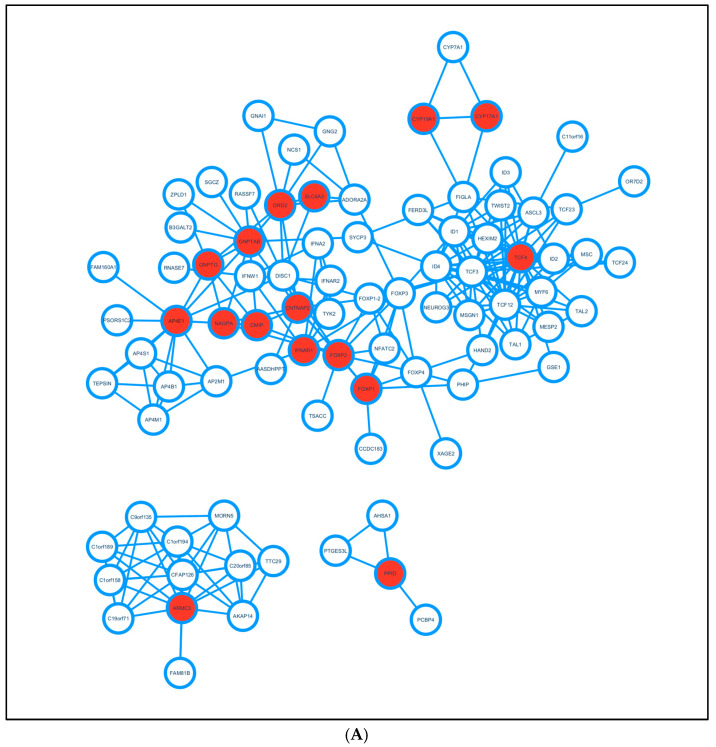

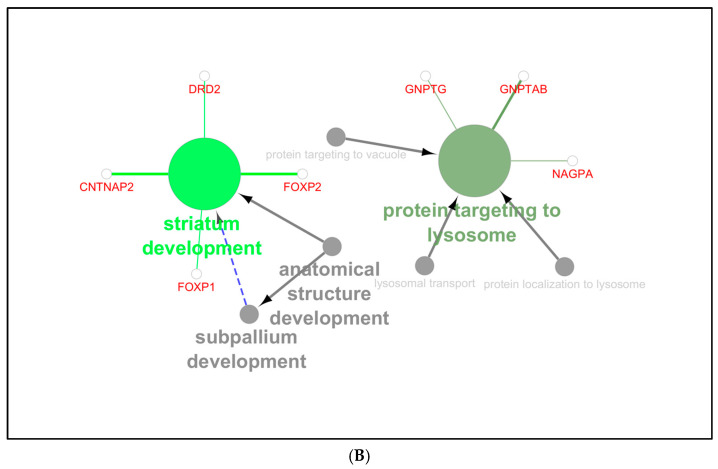
(**A**) Network of genes associated with language disorders: The PPI network was created using the STRING database, which contains all the protein interactions. The network was constructed with the collection of genes that were experimentally determined as interactions with a confidence score of 0.4 for the Homo sapiens species. The network was extracted to the Cytoscape platform, and the genes that were involved in the stuttering were annotated with a red color node to differentiate them from the others. (**B**) Functional analysis of genes: The functional analysis was performed for the network in (**A**) using the CLUGO plugin in Cytoscape to determine the role of these genes related to speech impairment.

Speech is defined as the ability to express one’s thoughts and feelings through articulate sounds, and it comprises three main components—articulation, voice, and fluency. Several aspects of speech can be understood, but in this section, we will focus on the genetic mechanisms of speech, explicitly stuttering [21,22,23]. A proper understanding of the genetic mechanisms of speech requires a thorough exploration of the genetic causes of certain speech disorders, such as verbal dyspraxia, speech language impairment, and stuttering. Although speech disorders are caused by several factors, which may be environmental or genetic, it is observed that, in several cases, by studying the genomes of people with speech disorders we can ascertain specific genes that majorly contribute to the presentation of these disorders. This is performed through linkage and association studies, which mark the approximate location of an etiologic genetic variant (such as an SNP) in a segment of DNA. Multiple genes are required for the normal functioning of the genetic mechanisms of speech (Table 1 and Table 2). A single gene or a group of genes participate in specific pathways that make speech and related functions possible, such as processing, understanding, formation, production, and fluency (Figure 2). Single-nucleotide polymorphisms (SNPs) are irregular base changes in DNA that mutate and can affect the functioning of genes in speech-related pathways. This can give rise to speech disorders. In this paper, only a few significant pathways are discussed in detail.

**Table 1:** The table summarizes the roles of key genes involved in speech and language development and their contributions to specific disorders.

Gene	Associated Disorder/Function	Chromosomal Location	Role in Speech and Language	Refs
*FOXP2*	Verbal Dyspraxia	Chromosome 7q	Regulates genes in brain regions for motor control, impacting speech and language. Disruption leads to speech deficits. Acts as a transcription factor, reducing neural gene expression.	[24]
*FOXP1*	Speech and Language Mechanisms	Chromosome 3	Involved in neural circuitry for speech and language development. Disruption causes speech delays and developmental issues.	[25]
*CNTNAP2*	Complex Language Impairment	Chromosome 7q35-q36.1	Encodes a neurexin protein for synapse function. Mutations linked to SLI and SSD. Works with *FOXP2* in gene-expression networks.	[26]
*GNPTAB*	Stuttering	Various chromosomes (2,3,5,7,9)	Involved in the lysosomal enzyme pathway. Mutations linked to stuttering.	[27]
*GNPTG*	Stuttering	Chromosome 7	Similar to *GNPTAB*, involved in the lysosomal enzyme pathway. Mutations linked to stuttering.	[27]
*NAGPA*	Stuttering	Chromosome 16p13.3	Involved in lysosomal enzyme targeting. Mutations contribute to stuttering.	[28]
*CMIP*	Specific Language Impairment (SLI) and Autism Spectrum Disorder (ASD)	Chromosome 16q23.2	Regulates phonological memory, critical for language acquisition. Linked to SLI and ASD.	[26]
*TCF4, STOX1A*	Combinatorial Gene-Expression Network in Speech	Chromosome 18q21.2, Chromosome 10q22.1	Involved in gene networks with *FOXP2* and *CNTNAP2* for speech and language development.	[1,26]
*DRD2*	Stuttering	Chromosome 11q23.2	Encodes dopamine receptor D2, linked to susceptibility to stuttering.	[29]
*SLC6A3*	Stuttering	Chromosome 5p15.33	Encodes dopamine transporter (DAT). Mutations affect speech and language, linked to stuttering.	[30]
CYP19A1	Neurodevelopmental Disorders	Chromosome 15q21.1	Involved in estrogen synthesis, potentially linked to neurodevelopmental speech disorders.	[31]
CYP17A1	Neurodevelopmental Processes	Chromosome 10q24.32	Influences steroid hormone biosynthesis. Role in speech disorders unclear.	[32]
PPID	Persistent Stuttering	Chromosome 4q33	Involved in protein folding. Mutations linked to stuttering by affecting brain development.	[33]
AP4E1	Neuroanatomical Anomalies	Chromosome 15q21.2	Mutations associated with brain anomalies in people who stutter.	[22]
IFNAR1	Developmental Stuttering	Chromosome 21q22.11	Mutations linked to stuttering in certain populations.	[34]
ARMC3	Persistent Stuttering	Chromosome 10p15.3	Associated with non-syndromic persistent stuttering in specific populations.	[35]

**Table 2:** The table summarizes the list of key genes involved in speech and language development for the functional gene analysis determined by CLUEGO.

Gene	Functions	Biological Functions	Pathophysiology	Refs
*FOXP1*	Encodes a transcription factor involved in brain development, particularly in areas related to language, cognition, and motor functions.	Striatum development, subpallium development, and anatomical structure development.	Impairment in striatum development affects the anterior and posterior language-processing networks.	[24,25,26,29,36]
*FOXP2*	Encodes a transcription factor that regulates genes involved in the development of neural circuits for speech and language.			
*CNTNAP2*	Encodes a protein involved in neuron–glia interactions, synaptic transmission, and neuronal migration.			
*DRD2*	Encodes a dopamine receptor involved in reward, learning, and motor control.			
*GNPTG*	Encodes a subunit of the enzyme N-acetylglucosamine-1-phosphotransferase, which is crucial for targeting lysosomal enzymes by adding mannose-6-phosphate markers.	Protein targeting to lysosome. Protein targeting to vacuole, lysosomal transport, and protein localization to lysosome.	Mutations in *NAGPA*, *GNPTG*, and *GNPTAB* have been associated with the speech disorder in Pakistani family members.	[27,28,37]
*GNPTAB*	Encodes the alpha and beta subunits of the enzyme N-acetyl glucosamine-1-phosphotransferase, essential for lysosomal enzyme targeting.			
*NAGPA*	Encodes an enzyme involved in the second step of mannose-6-phosphate marker formation, crucial for lysosomal enzyme targeting.			

## 4. Speech Disorders

Speech disorders encompass a broad range of conditions affecting an individual’s ability to produce sounds that result in fluent and clear speech vocabulary, syntax, morphology, semantics, or a combination of these elements of speech [38]. These disorders are heterogenous in nature and affect. These conditions can be congenital or acquired, and often present alongside various comorbidities, impacting quality of life and communication abilities.

**Figure 3:**
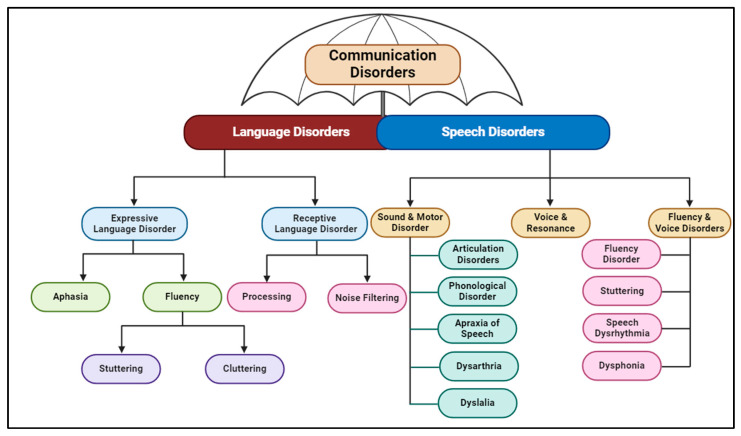
Hierarchical classification of communication disorders: The figure illustrates a broad classification of communication disorders, emphasizing the distinction and overlaps among subtypes (figure created using BioRender).

The American Speech-Language-Hearing Association (ASHA) classifies speech disorders into organic (caused by neurological, motor, anatomical, or sensory impairments) and functional (without a known cause). While many classification systems exist, most agree on overlapping error patterns in speech quality, voice, and fluency. This review adopted the following categories, shown in Figure 3.

### 4.1. Speech Sound Disorders

#### 4.1.1. Articulation Disorders

Articulation disorders involve atypical speech production through substitution, omission, addition, or distortion of sounds, often leading to unintelligible speech [39]. These disorders typically result from motor control issues in speech muscles, affecting sounds like /r/, /l/, and /s/, and are linked to atypical brain development in areas like the motor cortex and cerebellum [40]. In children, they manifest as mild-to-moderate speech defects and are often associated with hearing loss [41]. Prevalence ranges from 2.3% to 24.6% in school-aged children, with higher rates in boys and influenced by ethnicity. In adults, articulation disorders are linked to traumatic brain injuries, strokes, and neurological conditions, and often co-occur with other disorders, such as stuttering, ADHD, and language impairments [42]. Dyslalia refers to the misarticulation of phenomes, often due to learning errors or structural abnormalities, like cleft palate or dental issues. Neurobiologically, dyslalia is less about neural deficits and more about physical anomalies in the speech-producing organs. Poor oral habits, such as nail biting, thumb sucking, as well as atypical swallowing, have been ascribed as causing dyslalia, although with insufficient evidence to support the claim [39].

#### 4.1.2. Phonological Disorders

Phonological disorders involve difficulty learning a language’s sound system, leading to speech sound errors. This is linked to atypical neural processing in areas responsible for phonological representation and auditory processing. One theory suggests individuals with phonological disorders struggle to grasp the phonological rules of a language [40]. Researchers agree that early speech errors predict phonological disorders [43,44]. Another theory links phonological disorders to executive dysfunction, which affects higher cognitive functions, like reasoning, pattern recognition, and memory retention [45]. Impairments in executive functioning may result in errors in phonological speech production.

### 4.2. Motor Speech Disorders

Verbal speech production involves a sequence of motor coordination and executive functions. Motor speech disorders (MSD) are caused due to impairments in motor systems that result in speech production deficits. Impairments in motor systems can be due to neuromuscular dysfunction or disruption in higher-level motor commands [46]. The most commonly occurring motor speech disorders are dysarthria, speech disorders resulting from disrupted muscular control, and apraxia of speech, speech impairments due to disrupted motor planning [47].

#### 4.2.1. Apraxia of Speech

Apraxia of speech is a motor speech disorder in which individuals have difficulty planning and coordinating the movements necessary for speech [48]. Individuals with AOS often have trouble communicating what they want to say as result of faulty brain pathways in planning the sequence of speech sound production. It involves ‘effortful groping for articulatory movements’ in speech production [49]. The clinical presentation of apraxia is a slow rate of speech with consonants and vowel distortions, sound substitutions, and prosodic abnormalities [50]. Apraxia of speech can either be acquired or is present from birth. Acquired AOS, or neurological AOS, is predominantly a result of left-hemispheric strokes and neurodegenerative diseases. Childhood apraxia of speech is characterized by inconsistent speech production, vowel and voicing errors, consonant cluster deletions, and prosodic disruptions [51].

#### 4.2.2. Dysarthria

Dysarthria is a motor speech disorder resulting from neurological injury to the motor components of the speech production system. It results from disturbances in respiration, phonation, resonance, articulation, and prosody [52]. About 90% of individuals suffering from Parkinson’s disease [53] and 10–60% of individuals with a traumatic brain injury develop dysarthria [54]. There are several types of dysarthria, each associated with damage to different parts of the nervous system: flaccid, spastic, ataxic, hypokinetic, hyperkinetic, and mixed dysarthria.

### 4.3. Fluency Disorders

About 1% of children and adolescents in the global population suffer from stuttering, and even less from cluttering [55]. ASHA defines fluency as ‘continuity, smoothness, rate, and effort in speech production’. Therefore, interruption in the flow of speaking due to an atypical speech rate, rhythm of speaking, accompanied by irregular repetitions of words/syllables, and prolongation of sounds is classified as fluency disorder. Individuals suffering from fluency disorders have deficits in secondary mannerisms, such as speaking avoidance, anxiety, emotional and psychological distress, as well as avoidance of social situations that require speaking [56].

#### 4.3.1. Stuttering

Stuttering is characterized by a disrupted rate of speech, prolongation of sounds, difficulty in starting to speak, use of filler words, and repetitions. It is more common than cluttering in children and, in some cases, children outgrow it without any therapeutic interventions. Some degree of disfluency in speech is expected in all speakers, especially the repeated use of filler words. However, prolonged disfluency can be distressing to the individual, as they are self-aware of the condition, and it can lead to avoiding talking all together [57]. While idiopathic stuttering occurs due to functional or structural cerebral anomalies and is often limited to childhood, acquired stuttering occurs due to brain injury irrespective of age. In an extremely rare scenario, an individual can develop a psychogenic stuttering, usually after puberty, because of either an underlying psychiatric illness or psychological trauma [58].

#### 4.3.2. Cluttering

In contrast to stuttering, cluttering is characterized by rapid or irregular speech, with abnormal pauses, omission or contraction of syllables, and a dysregulated speech rhythm that is not typical of stuttering [59]. While there is repetition of speech, it is mostly repetition of words and parts of sentences, as opposed to sounds and syllables in stuttering. Cluttering is also characterized by monotonous speech, impaired word retrieval, and semantic–lexical impairments [55]. A key differentiation between stuttering and cluttering is that the latter is accompanied by poor spelling and writing abilities, which is intact in stuttering. Cluttering often occurs alongside auditory processing disorders, Tourette’s syndrome, ADHD, ASD, and learning disabilities.

### 4.4. Dysphonia or Voice Disorder

Dysphonia is a disorder characterized by difficulties in voice production, often due to problems with the vocal cords or the muscles controlling them. It is often called “hoarseness” and is an observable decline in voice quality [60]. Recent studies have shown an onset of dysphonia in individuals affected by COVID-19 [61].

#### 4.4.1. Mutism

Mutism is the inability or unwillingness to speak in certain situations despite having the ability to speak in others. It rarely occurs in isolation and is often accompanied by behavioral and psychological disturbances [62].

#### 4.4.2. Selective Mutism

It is the most commonly occurring mutism and is often associated with social anxiety disorder [63]. Research also shows that treating symptoms of anxiety through psychological interventions, such as cognitive behavioral therapy, has been effective in the long term [64].

#### 4.4.3. Cerebellar Mutism

This category of mutism often occurs post-posterior fossa tumor resection in children, but it can also occur due to vascular incidents and infections [65]. The clinical presentation is characterized by delayed onset (1–6 days after tumor resection) and a rapid and spontaneous recovery period (within 4 months) with speech dysarthria.

#### 4.4.4. Speech Dysrhythmia

Speech dysrhythmia involves disruptions in the rhythm and timing of speech, often seen in conditions like stuttering. Rhythm perception is crucial for synchronization of voice, movement, and emotion with whom we are interacting [66]. Clinical presentation of speech dysrhythmia is arrested speech, usually at the beginning of the sentence or word, and prolongation of words, often accompanied by stuttering.

#### 4.4.5. Childhood Speech Disorders

Childhood speech disorders encompass a range of speech impairments that occur during the developmental period. These can include any of the previously mentioned disorders but are often associated with developmental conditions, like autism spectrum disorder (ASD) or developmental language disorder (DLD). One in twenty preschool children show signs of developmental speech and language disorder stemming from learning and intellectual impairments aside from genetic contributions [67]. Childhood speech disorders are frequently linked to atypical development of language-related brain regions, such as the superior temporal gyrus and the arcuate fasciculus, a white matter tract that connects Broca’s and Wernicke’s areas [68].

#### 4.4.6. Broca’s Aphasia

Broca’s aphasia is one of the most commonly occurring expressive aphasia language impediments, caused by damage to the brain regions responsible for language. Although not a speech disorder, it often occurs with apraxia of speech and dysarthria. Broca’s aphasia results from damage to the brain region referred to as Broca’s area or the inferior frontal lobe of the dominant hemisphere, which is responsible for making sounds to create a word. One of the clinical presentations of Broca’s aphasia includes an inability to produce words, which affects the fluency of normal speech [69].

## 5. Psychological Comorbidities

Psychological comorbidities refer to the simultaneous presence of two or more psychological conditions in an individual. These comorbidities can complicate diagnosis and treatment, making it essential to explore their impact on overall health and treatment outcomes [70,71]. The dynamic nature of stuttering has led to various theories about its causes, with individuals often experiencing significant psychological distress. People who stutter frequently report feeling more comfortable speaking alone rather than in group settings. Common psychological issues associated with stuttering include fear of negative evaluation, heightened communication apprehension, and poor self-perception regarding communication competence. These factors can severely affect an individual’s quality of life [70,71,72,73]. Research has shown that adolescents and adults who stutter often face poorer psychosocial outcomes, including increased anxiety, particularly in those who have experienced childhood bullying [74,75]. Efforts have been made to explore the links between stuttering and various biopsychosocial factors, such as temperament, anxiety, depression, and ADHD. These studies aim to identify potential comorbid conditions in children, adolescents, and adults with stuttering [70,76].

Speech disorders encompass a wide range of challenges affecting an individual’s ability to communicate effectively, including difficulties with articulation, fluency, and voice resonance. These disorders often stem from complex interactions between neurological, physiological, and environmental factors, requiring comprehensive therapeutic interventions. However, speech impairments rarely exist in isolation—they frequently co-occur with psychological comorbidities that can significantly impact an individual’s emotional well-being and social functioning. Understanding the interplay between speech disorders and these psychological challenges is essential for providing holistic care and improving overall quality of life.

### 5.1. Social Factors of Stuttering and Its Comorbidities

#### 5.1.1. Environmental Factors

Cultural and societal norms are also suggested to affect stuttering. About 22.4% of Hispanic American college students believed that the pressure placed by parents on a child to speak two languages caused stuttering, and 39.4% agreed with the statement that switching from the L1 (Spanish) to the L2 (English) was the source of stuttering [76]. It was suggested that experience might interact with stuttering severity. Igbo children with negative school experiences stuttered more in English, the language of instruction, while those who faced challenges at home stuttered more in Igbo, their home language [77]. The parental dissatisfaction with their children’s “imperfect” Spanish, along with labeling common disfluencies in four Cuban American children, was the primary cause of stuttering. Environmental context reflects the influence of other people in the speaker’s life. The environmental context can influence the speaker via a conversation partner or, more generally, via society as a whole [56,78]. Studies suggest that speaking pressure, whether real or perceived, can have a negative impact on people who stutter [79,80].

One way that the impact of such factors on stuttering is explained is by the “demands and capacities” view. It proposes that disfluencies and stuttering occur when a child’s capacity for fluency is not the same as the speech performance demands placed on the child [81].

The concept to explain the different manifestations of demands and capacities has been discussed in a book by Starkweather. Demands can be both internal (e.g., an individual’s desire to express complex thoughts that require more sophisticated phonology, syntax, semantics, and pragmatic skills) and external (e.g., frequent interruptions and time pressure) [82]. The pressure to meet these external expectations can lead to heightened physical tension during speech, which may exacerbate stuttering severity and create additional challenges in communication [83,84].

One study proposed the view that failure to communicate and anticipating struggle contribute to the development of stuttering [85]. Failing to express oneself clearly or getting punished for disfluencies may result in tensing of muscles and fragmented speech. These behaviors, in turn, exacerbate communication troubles and intensify dread. Societal stigma, which often portrays stuttering as abnormal, reinforcing negative public opinions, adds to the issue. Self-stigma arises when individuals internalize negative societal expectations, such as the ideal of fluency, and view their stuttering as a personal shortcoming [86,87,88,89].

#### 5.1.2. Bilingualism

Early research showed that bilingual children exhibited more disfluencies in speech than their monolingual counterparts [90,91]. Bilinguals have been observed to perform worse than monolinguals on tasks requiring lexical access, such as naming objects [92], and verbal fluency tasks (e.g., coming up with as many words as possible for a given letter), irrespective of the dominant or non-dominant language being used. This has been attributed to the difference in vocabulary size with their monolingual peers in each of the two languages [93], as well as to the interference in lexical access between the languages [94,95]. In addition, early exposure to several languages could be a risk factor in stuttering [96]. As an extension, bilingualism was also suggested as a risk factor for developing stuttering in children [97].

On the flip side, other aspects of cognition, such as inhibitory control and task switching, improve [98,99]. These advantages are presumed to result from practice shifting from one language to another and inhibiting the language not used. Given that people with stuttering (PWS) may have reduced executive functions, a counterargument would be that bilingualism is a protective factor in children at risk for stuttering [100]. Such an effect has yet to be documented; however, it was hypothesized that bilingualism may act to offset deficits in executive functions that have been identified in numerous studies of monolingual PWS [101]. Other studies have shown no significant differences in executive functioning between monolingual and bilingual populations of school children [102,103]. There is yet to be a consensus among researchers as to whether bilingualism would act as a protective factor or a risk factor in the case of stuttering.

#### 5.1.3. Linguistic Factors

Various studies have suggested that differences in disfluencies across languages spoken by BWS can be attributed to language-specific variations in morphosyntax, phonology, and syllabic structure [104,105]. These claims are yet to be empirically tested and remain theoretical for now. While linguistic differences in areas like word formation, inflection, and sentence types [106] complicate comparisons of stuttering severity across languages, various studies have explored this notion of complexity. For instance, Brown et al. identified phoneme position, sentence position, grammatical class, and word length as factors contributing to higher stuttering frequency [107]. Other studies by Howell et al. and Ononiwu focused on phonological complexity between languages [108,109]. One notable method for assessing this complexity is the Index of Phonetic Complexity (IPC) scheme [110], which evaluates eight factors, such as consonant and vowel class, word shape, word length, and contiguous consonants. A study in 2007 and 2011 applied the IPC scheme to compare stuttering frequency in monolingual Spanish- and English-speaking individuals, revealing that English function words were more complex than Spanish ones [58,111]. In 2013, Al-Tamini et al. adapted this approach to Arabic with the development of an Arabic IPC (AIPC), finding that stuttered Arabic words were more phonetically complex than non-stuttered ones [112]. Another study in 2010 by Ononiwu extended the examination of phonological complexity across seven languages (Afrikaans, English, French, Igbo, Kannada, Mandarin, and Spanish) using various analytical methods, such as a four-factor phonological analysis and stress/rhythmic analysis, with English emerging as the most phonologically complex language based on these criteria [109]. Although these studies suggest that linguistic complexity may influence stuttering, the evidence remains largely correlational rather than causally proven. There is a need for more comprehensive experimental research to solidify these attributions and their practical applications. Investigating comorbidities in stuttering is crucial for several reasons. It enhances our understanding of the disorder’s presentation and the individual differences in experiences. This knowledge is essential for developing effective treatment plans tailored to the specific needs of individuals with stuttering and their comorbid conditions.

#### 5.1.4. Anxiety

Anxiety is a future-oriented emotion that is a long-acting response broadly focused on a diffuse threat, characterized by cognitive components of negative thoughts and beliefs of upcoming events, behavioral components of the desire to escape situations, and the physiological components of activation of the sympathetic nervous system [113,114]. It comprises a transitory state, known as the state anxiety, and a relatively permanent state, known as the trait anxiety, reflecting one’s personality characteristic of responding anxiously to potentially threatening situations [115]. Anxiety disorders, such as the generalized anxiety disorder, are diagnosed as a result of abnormally high levels of anxiety, such that the symptoms reflect the maladaptive behaviors of an individual, impacting their daily functioning in multiple contexts. More particularly, social anxiety disorder refers to the fear or avoidance of social interactions and situations that may result in scrutiny.

##### Link with Stuttering

Early research concluded the association between anxiety and stuttering to be weak [116]. This was also based on the difficulty in interpreting this link due to reasons such as the multidimensional nature of anxiety, small sample sizes in studies, differences in treatment status of patients based on when they were included in a study, and the measures used to assess anxiety [117].

i. Evidence from Children Who Stutter (CWS)

Studies show variable results for elevated anxiety among CWS. Prominent narrative reviews in this field suggest that CWS may not be predisposed to anxiety through increased familial risk or temperament traits, but they are at a higher risk of developing anxiety, as they are more likely to be exposed to negative peer reactions, bullying, and stereotyping during adolescence [72,114]. With limited results for pinning down a particular age for this, some studies indicate school age and teenage years as crucial periods for this development and manifestation of social anxiety—while negative attitudes toward speech develop at a young age, they worsen as they grow older [118,119]. CWS in school and adolescents with additional disorders also appear to be at a higher risk for developing social anxiety [120]. Comparatively, a recent meta-analysis by found a moderate summary effect size difference, indicating that children and adolescents who stutter present with increased anxiety symptoms (g = 0.42, *p* = 0.02, 95% CI (0.1, 0.743), df = 9.45) compared to their non-stuttering peers (presented statistic is the mean effect size difference, called ‘Hedge’s g’, for 851 participants, of whom 384 had a stutter, across the 11 studies considered). Upon performing a meta-regression analysis for situation-specific (state anxiety and social anxiety subscales) and general anxiety domains (trait anxiety and generalized anxiety subscales), they found that while elevated anxiety was observed in measures for both the domains of social and general anxiety, the measures used were not sufficiently sensitive to distinguish between the two, and rather a longitudinal approach would help determine the differences. It is important to note that elevated anxiety scores among CWS do not necessarily point toward clinical anxiety disorder but are mentioned in relation to differences in symptom summary scores [121]. However, the authors strongly suggested that the influence of recruitment bias accrued to clinically ascertained cohorts that make up over half of the included studies in the current review, eventually leading to the question of representativeness. Using a broader group of CWS from the population, including those who receive and those who do not receive clinical treatment, could lead to more generalizable findings.

ii. Evidence from Adults Who Stutter (AWS)

With some improvement in the methodological issues of studying links between anxiety and stuttering, meta-analyses have shown that persistent stuttering in adults is associated with significantly elevated trait and social anxiety when compared to non-stuttering adults [122]. Adults who stutter are shown to be at an increased risk of meeting the diagnostic criteria for clinical anxiety disorders, particularly social anxiety disorder [123,124]. It is supposed that increasing self-awareness and exposure to negative reactions from peers, especially due to increased demands of academic, vocational, social, and interpersonal aspects during adolescence and early adulthood, lead to anxiety in AWS [114].

An interesting hypothesis about the link between the motor symptoms of stuttering and anxiety shows the unidirectional relationship between the two. While reducing anxiety may not work toward improving fluency amongst AWS, improvement in fluency may result in reduced anxiety [85,125,126]. However, there have been mixed results for the latter [127,128].

#### 5.1.5. Depression

The American Psychological Association characterizes depression as a prolonged sad, empty, or irritable mood along with somatic and cognitive changes that have a substantial impact functionally.

##### Link with Stuttering

There have been mixed findings regarding elevated depression symptoms in some adolescents and young adults who stutter, such that some found differences compared to controls [129,130] and some did not [131]. The differences in depression scores that were high among the stuttering group for four out of the five measures reported across three studies, but the difference was not statistically significant in any study. They attributed this ambiguous result to the age range of the samples across studies (<15 years) given the later onset of depression in the general population [121].

#### 5.1.6. Temperament

Temperament is referred to as the biologically determined part of personality that is assumed to be constitutional, inherent, and relatively stable [132]. It could also be described as the individual differences in one’s emotion-based habit patterns [133].

##### Link with Stuttering

Research in this domain regarding stuttering has given rise to the assumption that people with this condition tend to have a reactive temperament—that is, they are more emotionally sensitive—which interacts with their linguistic or motor problems of stuttering. It is proposed that these traits can increase the risk of developing chronic stuttering, as it is believed that people with stutter may react with a stronger muscular tension to emotional problems [134]. Children with stutter who experience greater negative reactivity are also more likely to develop anxiety [135]. Theoretically, it has been proposed that young children with a genetic predisposition to stuttering with typical temperamental tendencies of increased negative affect, and reduced emotional and attentional regulation, may be likely to start stuttering, with an increased risk of developing negative reactions to stuttering and reduced resilience to coping in the long term [134,136]. However, in a study published by Van Riper et. al., they did not find any substantial differences in the personalities of people who stutter [137].

i. Inconsistent findings using different measures

The evidence from parent-report measures and experimental tasks has not been able to corroborate the relationship between stuttering severity and/or frequency with temperamental traits in CWS using these two measures [72]. The experimental studies with CWS under seven years have generally been able to find a relationship between temperament domains of emotional reactivity and regulation and stuttering severity and/or frequency [138]. On the other hand, parent-report measures of temperament with this age group showed inconsistent results [139,140,141,142,143].

The possibility of a subgroup of children who stutter (CWS) exhibiting traits of hyperactivity and impulsivity, which may result in lower scores for shyness. This suggestion is based on findings that CWS display significantly lower perceptual sensitivity (possibly indicating inattention), reduced inhibitory control, poorer attentional shifting, and higher activity levels compared to children who do not stutter (CWNS). Additionally, some results, though not statistically significant, suggested that certain CWS may show lower anxiety traits than CWNS [139]. The varied findings are a result of methodological differences, such as the use of small sample sizes in some studies, varied age ranges included, and the kind of sample studied (community, clinical, or research). Differences in investigating stuttering frequency or severity along with the measures used to assess them also add to the mixed literature [144].

ii. Recent Evidence

Parent and child perceptions of temperament were both considered to determine its role in stuttering frequency and impact in children under the age of seven years. While negative reactivity was associated with stuttering’s impact in CWS regardless of age from a parent’s perspective, it was not the case when children reported their attitudes toward communication. Positive reactivity and emotional regulation were not associated with stuttering’s impact in young CWS from both perspectives. Temperament was not associated with stuttering frequency [144].

#### 5.1.7. Attention Deficit Hyperactivity Disorder (ADHD)

Attention deficit hyperactivity disorder (ADHD) is characterized as a neurodevelopmental disorder, with a combination of persistent behaviors of inattention, impulsivity, and hyperactivity, that begins in childhood (National Institute of Mental Health). The fifth edition of the Diagnostic and Statistical Manual of Mental Disorders (DSM-5) defines inattention and disorganization as the inability to stay on task and listening issues that are not in accordance with one’s age or developmental level [145]. Hyperactivity and impulsivity can be indicated by overactivity, fidgeting, difficulty in staying seated, intruding into other people’s activities, and inability to wait. ADHD is diagnosed in a person when their attention difficulties or hyperactivity exceed what is generally seen in their equivalent mental age group. People with this disorder can exhibit a combined presentation—predominantly on attention or predominantly on hyperactivity and impulsivity.

##### Link with Stuttering

The commonality between people who stutter and people who have ADHD is confirmed by evidence of attention issues and speech disfluencies occurring in both these conditions, highlighting the fact that they may share similar psychological, social, and neural issues [146,147,148,149,150,151,152,153].

i. Evidence from Behavioral Measures in CWS

Parent-reported surveys: Studies using tools like the Children’s Behavior Questionnaire and the Behavioral Style Questionnaire consistently found significant differences between CWS and CWNS in ADHD-related traits. CWS exhibit several traits that overlap with characteristics commonly associated with ADHD. These include lower perceptual sensitivity and poor adaptability, potentially indicating inattention [139,154]. Additionally, CWS demonstrate lower inhibitory control and poorer attentional shifting, which are core ADHD traits [155]. A higher activity level is also observed in CWS, aligning with hyperactive tendencies seen in ADHD [139]. Furthermore, CWS show lower emotional regulation and heightened emotional reactivity, characteristics that similarly overlap with ADHD profiles [156,157].

ii. Experimental Evidence

Experimental studies supported the parent-reported findings, with some CWS showing a tendency toward impulsivity and lower inhibitory control. This suggests that a subset of CWS may have traits consistent with ADHD, though not all cases of stuttering involve these traits [158,159].

iii. Genetic and Neurological Factors

Some studies suggest that ADHD traits in CWS may have a genetic or neurological basis. CWS with neurological incidents linked to ADHD traits might experience more persistent stuttering, which could contribute to a higher incidence of adults who stutter (AWS) [146]. These findings indicate that the ADHD-like traits in CWS may be part of a broader neurodevelopmental profile.

iv. Association with Cluttering

The speech disorder of cluttering, often associated with ADHD-like tendencies, such as impulsivity and disorganized speech, may provide another explanation for the link between ADHD traits and stuttering [152]. This suggests that cluttering and stuttering may sometimes overlap, further complicating the relationship between stuttering and ADHD.

v. Prognostic Value of ADHD Traits

Recovery from stuttering: Interestingly, some studies suggest that hyperactivity and lower emotional reactivity may serve as positive prognostic factors in childhood stuttering. Children with these traits might have a higher likelihood of recovering from stuttering compared to those who do not exhibit these traits [149,160]. This offers a nuanced view of ADHD-like traits, indicating they may not always worsen stuttering outcomes.

vi. Evidence from Network Modeling

While both stuttering and ADHD conditions are said to affect working memory, more nuanced details are required to check their similarities in cognitive architecture [161,162,163,164]. Kazazi et al. probed into newer and more contemporary research methods to determine the link between the two disorders [165]. They suggested the use of network modeling, which is used in studies of comorbidities amongst disorders, wherein they are viewed as clusters of directly related symptoms that can interact with one another and be supposedly causally linked [166,167,168]. These networks of symptoms are then used to find and analyze statistical relationship patterns in multivariate psychological data [169]. Using this method, they also suggested that symptoms of stuttering and ADHD, while impacting the frontal cortex, are not comorbid, meaning their cognitive architectures may be different. While working memory and executive function are impaired in both these disorders, they affect different types of attention in both. Inattention in people with stuttering issues can be assessed better using an auditory selective attention task, while the same can be assessed better using sustained attention tasks in people with ADHD. They proposed the use of network models so that clinicians can better assess these conditions and provide new treatment strategies [165].

#### 5.1.8. Autism Spectrum Disorder (ASD)

Autism spectrum disorder is a neurodevelopmental disorder with a higher prevalence in males, characterized by persistent difficulties in social interaction, communication (verbal and nonverbal), and a compulsion of restricted and repetitive behaviors [170,171]. It is also characterized by knowledge or specialization regarding a particular skill or topic, which may be exhibited to differing lengths [172].

##### Link with Stuttering

The developmental disorders of ASD and stuttering share many similarities. Both these disorders are a part of a class of disorders that develop due to anomalies in cerebral lateralization [173]. In the United States, the prevalence of stuttering alone in children rests at 5%, while an estimated prevalence of both stuttering and ASD would lie at around 0.04% [174].

i. Heritability

In terms of heritability, stuttering and ASD both seem to show a significant inclination toward the nature, such that around 60% of the variance in the onset of stuttering can be explained by genetic factors, and the genetic contribution in autism can explain anywhere from 64% to 91% [175]. While it is believed that there could be multiple genes responsible for the origin of autism, recent evidence using modern genetic testing tools suggests that mutation in the ARIH2 gene could play a key role in causing neurodevelopmental disorders, such as ASD and intellectual disability [176,177].

ii. Onset

Both stuttering and ASD develop well after birth, such that their onset cannot be determined or prevented at birth [178]. However, autism symptoms develop within the first year of life, while the onset of stuttering occurs in the second, third, or even several years after that [179].

iii. Symptom Differences

A recent study found qualitative differences in stuttering behaviors between a group of children with stuttering and children in the autism spectrum group. Word-medial and word-final disfluencies atypical to the stuttering group were exhibited several times in the ASD group [180].

#### 5.1.9. Intellectual Disability

Intellectual disability (ID) is a neurodevelopmental condition that encompasses deficits to an individual’s adaptative and intellectual functioning [181]. Intellectual functioning incorporates the characteristics of intelligence, the abilities assessed by standardized intelligence tests, and the consensus view that intellectual functioning is influenced by other human functioning dimensions and by systems of supports. Adaptive behavior is the collection of conceptual, social, and practical skills that have been learned and are performed by people in their everyday lives. Adaptive functioning serves as the basis for categorizing ID into mild, moderate, severe, and profound. ID affects approximately 1–3% of the population [182].

The presence of stuttering was associated with higher odds of intellectual disability (odds ratio (OR) = 6.67, *p* < 0.001) [129]. It has been suggested that impaired intellectual ability could account for a substantial number of children with speech and language delays due to the correlation between language and other intellectual deficits [183]. The percentage of ID in the people with communication disorders population in Utah was approximately triple that of the mental retardation rate in the general 8-year-old population in Atlanta in 2000. However, if the prevalence of communication disorders was adjusted to exclude all cases with an IQ of 70 or below, the overall communication disorders prevalence rate would not be significantly decreased (6.35% to 6.14%) [184,185].

## 6. Conclusions

The relationship of stuttering with temperament, anxiety, depression, and neurodevelopmental disorders, such as ADHD, ASD, and ID, has a complex nature. However, examining its comorbidities is essential to understanding the conditions that frequently coexist with stuttering [186]. Current literature suggests the use of a better methodology to probe into these comorbidities [117,144]. However, research about these links has helped zoom into the nuances of experiences of people who stutter. Recent evidence suggests that higher negative reactivity scores and lower positive reactivity and self-regulation scores are associated with elevated levels of anxiety and depression in those who stutter, showing an interrelation among temperament, anxiety, and depression. Temperament and ADHD have also been linked, suggesting that emotional reactivity and hyperactivity in CWS could potentially be positive prognostic factors [149,155]. Newer methodologies, such as network modeling, virtual reality, and a combination of cognitive, behavioral, and physiological assessments, may help us delve deeper into the relationship between stuttering and its comorbidities [165].

## 7. Future Outlook

The future of research and treatment in speech disorders is set to evolve with advancements in neuroscience, genetics, digital, and machine learning technologies. The intersection of these technologies will help map out the intricate neural circuits that underlie speech production, comprehension, and motor control with greater precision. By identifying specific neural pathways disrupted in speech disorders, targeted interventions that address the underlying neural dysfunctions have the potential to transform methods in speech therapy. As genetic testing becomes more affordable and accessible, personalized genetic profiling can enable clinicians to diagnose speech disorders at an earlier stage and tailor interventions to a patient’s specific genetic makeup. Gene therapy for genetic hearing disorders are in clinical trials and can also be applied to speech disorders by targeting and correcting the specific genetic abnormalities. The integration of digital therapies combined with machine-learning-based speech recognition tools can be applied to personalized therapy programs that adjust to the patient’s needs in real time. Music-based therapies are already mainstream therapies. The NIH has allocated significant funds for research on the use of music therapies, spearheaded by the ex-director, Francis Collins. The use of rhythm, timing, and melody in music therapy is already being applied to speech production and motor coordination, helping patients—particularly those with fluency disorders like stuttering—overcome disfluencies. The role of psychological treatment in managing speech disorders focuses on integrated care models, where speech/language pathologists, psychologists, and other mental health professionals address the emotional and psychological components of speech disorders. This holistic approach will help reduce the stigma associated with speech disorders and provide patients with more comprehensive care, improving outcomes in both speech fluency and mental well-being. In conclusion, the future of speech disorder treatment is headed toward more individualized, multidisciplinary, and technologically advanced solutions. By harnessing the power of genetics, neuroscience, digital innovation, and psychological care, the field will move closer to offering patients more effective, precise, and accessible treatments tailored to their unique needs.
